# Optimization of the Cryoprotectants for Direct Vat Set Starters in Sichuan Paocai Using Response Surface Methodology

**DOI:** 10.3390/foods14020157

**Published:** 2025-01-07

**Authors:** Lianqun Wu, Zhenying Yang, Ying Zhang, Ling Li, Chunli Tan, Lixia Pan, Yanping Wu, Kai Zhong, Hong Gao

**Affiliations:** 1 College of Biomass Science and Engineering, Sichuan University, Chengdu 610065, China; wulianqun@stu.scu.edu.cn (L.W.); lilingscu2022@163.com (L.L.); 2022141500141@stu.scu.cn (C.T.); wyp9202@163.com (Y.W.); gao523@hotmail.com (H.G.); 2 Sichuan Teway Food Group Co., Ltd., Chengdu 610000, China; yzy2508@163.com; 3 Guangxi Light Industry Science and Technology Research Institute Co., Ltd., Nanning 530031, China; 002tweety@163.com; 4 National Key Laboratory of Non-Food Biomass Energy Technology, Guangxi Academy of Sciences, Nanning 530007, China; panlixia@gxas.cn; 5 Guangxi Key Laboratory of Marine Natural Products and Combinatorial Biosynthesis Chemistry, Guangxi Academy of Sciences, Nanning 530007, China

**Keywords:** cryoprotectant, vacuum freeze-drying, direct vat set starter, *Lactiplantibacillus plantarum*, *Bacillus subtilis*, Sichuan paocai

## Abstract

The quality of Sichuan paocai in natural fermentation is often inconsistent due to the complexity of its microbial community and environmental influences. To address this, dominant microbial strains were selectively inoculated to improve the product’s quality and safety. However, vacuum freeze-drying, commonly used to prepare direct vat set (DVS) starters, can significantly damage strains due to freezing stress. This study aimed to optimize a freeze-drying protection system for *Lactiplantibacillus plantarum* and *Bacillus subtilis* to enhance their survival. Using response surface methodology, combinations of cryoprotectants were evaluated. A formulation comprising skim milk powder, glycerol, sucrose, and L-proline significantly improved strain viability after lyophilization, outperforming single cryoprotectants. Further investigation into storage conditions revealed that low temperatures (−20 °C) provided the best preservation for DVS starters. Furthermore, the optimized DVS starters demonstrated excellent fermentation performance in Sichuan paocai, enhancing its color, flavor, and sensory quality compared to natural fermentation. These findings offer a reliable freeze-drying protection strategy for survival and viability of *L. plantarum* and *B. subtilis*.

## 1. Introduction

Sichuan paocai (SCP), known for its tangy taste, is popular as a daily side dish [1], and its production helps balance vegetable supply, fosters unique regional brands, and supports Chinese food culture and the national economy [2,3]. SCP is a traditional fermented vegetable dish made by immersing fresh vegetables in a salt solution with spices. The fermentation process of SCP is driven by lactic acid bacteria (LAB), mainly *Weissella* and *Lactobacillus*, which convert complex compounds into simpler, more nutritious forms, reducing anti-nutritional factors like tannins and phytates [4]. This microbial activity enhances the nutritional value of SCP and contributes to its preservation. However, fresh vegetables carry a diverse microbiota, and some microorganisms can degrade the quality of SCP by producing nitrites and unpleasant odors, risking consumer safety [5]. Furthermore, natural fermentation in SCP is highly temperature-sensitive, thus complicating efforts toward large-scale production [6]. Therefore, it is critical to control the fermentation process and improve the stability of the fermentation for maintaining the quality of SCP products.

To stabilize the SCP fermentation process, researchers have explored inoculated fermentation to shorten fermentation time and reduce undesirable compounds like nitrite. For instance, back slopping inoculated fermentation, the practice of using aged brine, has been attempted, but it has limitations due to its inconsistency and lack of stability, and its final product showed poor chewiness [7,8]. Studies have thus turned to direct inoculation of LAB strains, like *Lactobacillus casei* and *Saccharomyces cerevisiae*, to improve the texture of SCP, reduce nitrites, and enhance sensory qualities [9,10]. However, these inoculations have not fully replicated the rich flavor profile of naturally fermented SCP [11]. More recently, non-LAB microorganisms have shown potential in SCP fermentation. For example, inoculation with *Bacillus megaterium* L222 could significantly increase the number of *Weissella* and reduce nitrite and sulfide levels in SCP products [12]. Inoculated *Bacillus cereus* GW-01 to ferment SCP could degrade pyrethroid residues in vegetables, increase the number of LAB, and improve the quality of the product [13]. Additionally, in our previous study, inoculation with *Bacillus subtilis* Y61 improved Sichuan paocai quality and increased the abundance of LAB during the SCP fermentation [14], suggesting the potential of combining LAB and non-LAB strains in the paocai industry.

Direct vat set (DVS) starters, highly concentrated strains that can be added directly to substrates without pre-culture, offer simplicity, high cell viability, and cost-effectiveness [15]. DVS starters are widely used in the fermentation industry to obtain high-quality products, such as fermented beef [16], high-ethanol wines [17], and vegetable beverages [18]. Nevertheless, the DVS starters associated with SCP in the available studies contained only LAB, and the effects of DVS starters prepared via vacuum freeze-drying on the viability and vigor of the strains were still lacking [9]. Importantly, during freeze-drying, ice crystal formation can damage cell membranes, reducing bacterial survival. Cryoprotectants such as skim milk powder, glycerol, and sucrose can mitigate this by controlling ice crystal growth, and composite cryoprotectants often perform better than single protectants [19,20]. Therefore, it is critical to optimize the protectants and process of DVS starters for guaranteeing the variability of strains.

To address the aforementioned problems and support industrial-scale SCP production, we selected *B. subtilis* Y61 and *L. plantarum* to develop direct vat set starters based on our previous findings [14] via single-factor experiments (SFE) and response surface methodology (RSM). RSM is a statistical technique [21] that uses mathematical and statistical models to optimize process variables by analyzing the effects of multiple factors on the response variable, ultimately finding the optimal operating conditions. It overcomes the limitations of orthogonal experiments without precise mathematical models, and it is more suitable for in-depth quantitative analysis and optimization predictions [22]. RSM has been successfully applied in various fields, such as in optimizing the extraction process of protein fractions from cashew apple bagasse [23], enhancing the content of aglycone isoflavones in soymilk through incubation condition optimization [24], and designing freeze-dried protectants for starter culture preparation in winemaking [17]. Hence, after we used SFE to evaluate the effects of four cryoprotectants on post-freeze-drying viability, RSM was applied to refine the optimal mixture for maximizing viable counts. Finally, we assessed its stability under three storage temperatures and applied the DVS starters to SCP fermentation. This study will advance the development of high-viability DVS starters for SCP, supporting quality consistency in large-scale SCP production.

This study aims to optimize the freeze-drying protection approach for *L. plantarum* and *B. subtilis* using RSM, with the goal of improving cell viability and fermentation performance. The results of this study are expected to facilitate the application of the DVS starters in the industrial production of stable and high-quality Sichuan paocai.

## 2. Materials and Methods

### 2.1. Materials and Reagents

Man, Rogosa, and Sharpe (MRS) agar medium and nutrient broth (NB) were purchased from Qingdao Hi-Tech Industrial Park HaiBo Biotechnology Co., Ltd. (Qingdao, Shandong Province, China). MRS broth and nutrient agar medium (NA) were purchased from Hangzhou Microbiology Reagent Co., Ltd. (Hangzhou, Zhejiang Province, China). Sodium hydroxide, sodium tetraborate (pentahydrate), potassium ferricyanide (trihydrate), zinc acetate (dihydrate), anhydrous p-amino benzene sulfonic acid, α-naphthylethylenediamine dihydrochloride, sodium nitrite, glacial acetic acid, hydrochloric acid, glycerol, sucrose, and L-proline were purchased from Chengdu Colony Chemicals Co. (Chengdu, Sichuan Province, China). Skim milk powder was purchased from FeiJing Biotechnology Co., Ltd. (Fuzhou, Fujian Province, China). Radish, ginger, garlic, red chili peppers, peppercorns, salt, rock sugar, and white wine used for fermenting Sichuan paocai were purchased from the local market in Chengdu, Sichuan Province, China.

### 2.2. Bacterial Strains and Activated Culture

*Lactiplantibacillus plantarum* SICC 1.1418 was purchased from the Sichuan Microbial Resource Platform Strain Preservation Center and was preserved in glycerol at −80 °C in a ultralow temperature freezer (Haier Group Co., Qingdao, China) after expanding the culture in our laboratory. *Bacillus subtilis* Y61 was screened in a jar of high-quality Sichuan paocai and preserved in glycerol at −80 °C in our laboratory.

*L. plantarum* and *B. subtilis* were firstly streaked and cultured using MRS solid medium and NA medium, respectively, and placed in a constant temperature and humidity incubator at 37 °C for 24 h. Single colonies on the plates were taken using an inoculation loop and inoculated into MRS broth and NB broth, respectively, and incubated for 12 h at 37 °C on a shaker (Taicang Hualida Experimental Equipment Co., Ltd., Taicang, China, HZ-9210K). The bacterial solution was taken with an inoculating loop and streaked in MRS solid medium and NA medium again, then stored at 37 °C in a constant temperature and humidity incubator (Shanghai Shenxian Thermostatic Equipment Factory, Shanghai, China, HSX-100CL) for subsequent experiments.

### 2.3. Preparation Procedure and Determination of Survival Rate of Direct Vat Set Starter

Single colonies of *L. plantarum* and *B. subtilis* grown on solid medium were inoculated in MRS broth and NB broth, respectively, and incubated on a shaker at 37 °C and 120 r/min for 19 h to obtain their respective initial cultures. Then, 4% of the culture solution was inoculated in new MRS broth and NB broth, respectively, and cultured for 19 h to obtain the enriched culture solution of the two strains at the late logarithmic growth stage. Then, the cultures were centrifuged at 8000 r/min for 15 min at 4 °C in a centrifuge (Shanghai Luxiang Instrument Centrifuge Instrument Co., Ltd., Shanghai, China, H17.5R), and the supernatant was discarded to obtain the bacterial sludge. The bacterial slime was washed three times with saline and dissolved to obtain the bacterial suspension, which was homogeneously mixed with the cryoprotectants in a freeze-drying vial in a ratio of 1:3, with an equal volume of saline used as the control. After being pre-frozen for 4 h at −80 °C, the samples were quickly placed in a vacuum freeze dryer (Ningbo Xinzhi Bio-technology Co., Ltd., Ningbo, China, SCIENTZ-10N) with a cold trap temperature of −64 °C, a vacuum degree of 11 Pa, and a freeze-drying time of 48 h. The incubation time of the two strains was determined by growth graphs (Figure S1).

Saline was added to the DVS starters and recovered to the original pre-lyophilization volume, which was diluted appropriately for 3 gradients coated for counting. The formula for the survival rate of the DVS starters was calculated as follows:

survival rate (%) = N_1_/N_0_ × 100% (1)

where N_1_ is the number of viable bacteria per unit volume of DVS starters, and N_0_ is the number of viable bacteria per unit volume of the bacterial solution before freeze-drying.

### 2.4. Single-Factor Experiment

Five gradients (gradients 1, 2, 3, 4, and 5) and a blank control (gradient 0, with an equal volume of saline added) were set for each of the four cryoprotectant additions as independent variables, referring to Table 1. The five gradient concentrations of skim milk powder were 40, 80, 120, 160, and 200 mg/mL, and the five gradient concentrations of sucrose, glycerol, and L-proline were 20, 40, 60, 80, and 100 mg/mL, respectively. The DVS starter was prepared and the survival rate was determined according to the experimental method described previously.

### 2.5. Response Surface Methodology

A response surface experiment with three factors and three levels was constructed based on the results of the single-factor experiment. The design and results are elaborated in Table 2.

The cryoprotectants to be optimized for *L. plantarum* were skim milk powder (A_1_), sucrose (B_1_), and L-proline (C_1_). For *B. subtilis*, the other three cryoprotectants—skim milk powder (A_2_), sucrose (B_2_), and glycerol (C_2_)—had better protection effects than L-proline. Therefore, these were selected as the factors for optimization. Meanwhile, based on the results of the single-factor experiment, L-proline (60 mg/mL) was selected as a fixed factor for *B. subtilis*. Design Expert 8.0.6, in conjunction with Box–Behnken design, was used in this experiment to find the optimal response by fitting the data to a quadratic curve using lyophilized viability as the response value, with one-half of the concentration gradient in the single-factor experimental between high level, low level, and the center of significance.

### 2.6. Verification Experiment

Based on the quadratic polynomial model obtained from the response surface experiment, the maximum value of the DVS starter survival rate was taken as the optimization objective to obtain the cryoprotectant formulations of the two strains. The optimized scheme was used to prepare the DVS starter, and the model was validated by calculating its freeze-drying survival rate.

### 2.7. Determination of Temperature on the Survival Rate of the Direct Vat Set Starters During Storage

To investigate the stability of the DVS starters under different storage temperatures, Three temperatures were chosen to represent the typical storage conditions encountered in practical applications and research. Specifically, 25 °C simulates ambient storage conditions that are common in non-refrigerated environments; 4 °C reflects the refrigeration conditions widely used for short-term storage and transportation, ensuring that microbial activity is kept at a low rate; −20 °C is the frozen storage condition and is often used for long-term preservation to minimize cellular metabolic activity and extend viability [25]. *L. plantarum* and *B. subtilis* were vacuum-sealed and stored at the three temperatures. The survival rates of the DVS starters were determined on the 15th and 30th day of storage compared with those of the initial DVS starters.

### 2.8. Application of Direct Vat Set Starter in Sichuan Paocai

#### 2.8.1. Preparation of Sichuan Paocai

The enriched cultures of *L. plantarum* and *B. subtilis* at the late logarithmic growth stage were mixed at a ratio of 1:3 (*V*/*V*) to obtain 60 mL of bacterial suspension after adjusting the suspension to 10^8^ CFU/mL using saline. The inoculation ratio of the two strains, which was determined pre-experimentation, resulted in Sichuan paocai with optimal physicochemical indexes and sensory evaluations (Figure S1). After centrifugation at 8000 r/min at 4 °C for 15 min, the supernatant was discarded to obtain the bacterial slurry, which was dissolved in 5 mL of saline as the inoculum.

Radishes were washed and sliced into rounds about 2 mm thick. In a paocai jar with a capacity of 5 L, 2 L of boiled and cooled drinking water was added, in which 6% (*m*/*v*) salt, 1% (*m*/*v*) rock sugar, and 3% (*V*/*V*) white wine were dissolved. Then, 50% (*m*/*v*) radish slices, 0.2% (*m*/*v*) peppercorns, and 3% (*m*/*v*) ginger, garlic, and red chili pepper were added. The natural fermentation group (NFP), the inoculated fermentation group (SFP), and the DVS starter fermentation group (DFP) were set up by adding 5 mL of saline, 5 mL of the above inoculum, and 5 mL of saline-dissolved DVS starters of equal cellular volume, respectively. The altar was covered, water-sealed with ultrapure water, and then fermented at 25 °C for 7 consecutive days. Samples of the paocai liquid were taken at the same time every day to determine the physicochemical indexes.

#### 2.8.2. Determination of Physicochemical Indexes and Sensory Evaluation

The pH and nitrite content of the Sichuan paocai was determined according to previous studies [12]. Texture analysis (TPA) was performed based on the hardness and chewiness of the paocai on the seventh day of fermentation; the probe and setup parameters are described in the previous literature [26]. In addition, chromaticity color difference measurements were performed (CM-5, Konica, Tokyo, Japan), where L*, a*, b*, and Δ*E* denote the luminance, red–green difference, yellow–green difference, and total color difference, respectively. The total color difference was calculated as follows [27]:

(2) $$\Delta E=\sqrt{{\left(L_{0}-L\right)}^{2}+{\left(a_{0}-a\right)}^{2}+{\left(b_{0}-b\right)}^{2}}$$

Finally, the Sichuan paocai on the fourth and seventh days was evaluated in terms of color, aroma, taste, and texture. The detailed evaluation criteria are shown in Table S1. It should be noted that each subject provided informed consent prior to participating in the study.

### 2.9. Statistical Analysis

All experiments were performed at least three times unless otherwise stated. The data were first processed using Excel 2019 and then analyzed via one-way ANOVA and Duncan’s test using SPSS (version 22.0, Chicago, IL, USA) to determine significant differences (*p* < 0.05, *n* = 3) due to their reliability and ability to ensure accurate comparisons. A one-way ANOVA, a well-established method for determining significant differences among group means [28], is particularly suitable for analyzing the single independent variables involved in this study with multiple levels, while Duncan’s test was applied as a post hoc method to clarify specific group differences [29]. Furthermore, the results of the experiments were plotted using Origin 2024.

## 3. Results and Discussion

### 3.1. Analysis of Single-Factor Experiments to Determine the Optimum Concentration of Different Cryoprotectants

**Figure 1:**
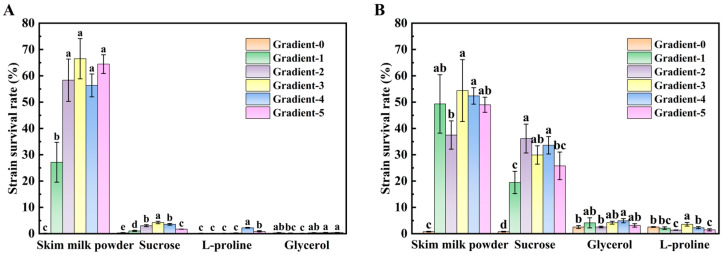
Effects of different kinds and different gradient concentrations of protectants on the survival rate of cryoprotectants of *L. plantarum* (**A**) and *B. subtilis* (**B**). For each gradient concentration of the same kind of cryoprotectant, the values corresponding to different letters are significantly different (*p* < 0.05).

Figure 1 illustrates the relationship between various types and concentrations of cryoprotectants and the survival rates of the strains. Skim milk powder, sucrose, and L-proline all enhanced the survival of *B. subtilis* and *L. plantarum*, while glycerol did not significantly protect *L. plantarum*. This highlighted that the same cryoprotectant had varying protective effects depending on the microorganism. Among the tested cryoprotectants, skim milk powder, a non-permeable protectant, provided the best protection. At a concentration of 120 mg/mL, survival rates of 54.38% for *B. subtilis* and 66.51% for *L. plantarum* were achieved, consistent with findings in existing studies [30]. Previous studies have shown that skim milk powder offers excellent protection for other strains, such as *Lactobacillus curvatus*, during freeze-drying [31]. The reason may be that skim milk powder, as a non-permeable macromolecular protectant, can utilize hydrophilicity to form a stable water molecule layer, hindering the transfer of bound water out of the cell and slowing down the death of the cell due to water loss [32].

The protective effects of sucrose and L-proline on *L. plantarum* initially increased with concentration, reached a peak, and then declined at higher concentrations (Figure 1A). The optimum concentrations for protection were 60 mg/mL for sucrose and 80 mg/mL for L-proline. For *B. subtilis*, the optimal concentrations of sucrose and L-proline were 40 mg/mL and 80 mg/mL, respectively. Sucrose and L-proline, classified as semi-permeable cryoprotectants, can penetrate the cell wall, increase viscosity, and form a buffer layer between the cell wall and membrane. This buffer layer reduced ice crystal formation outside the cell membrane, thereby minimizing damage to the cell membrane and increasing cell survival [33,34].

For glycerol, the protection of *L. plantarum* via the addition of glycerol was comparable to that of the control group (Figure 1A). Glycerol, a typical osmotic protectant, can replace tightly bound water molecules to prevent excessive dehydration and protein denaturation during the drying process. And, it also inhibits large ice crystal formation inside the cell. However, glycerol contains a high percentage of unfrozen water (up to 45%), which could lead to membrane damage from extracellular ice crystals when used alone, explaining why the *L. plantarum* was less protected. On the other hand, the *B. subtilis*, which can form spores under adverse conditions, showed improved survival when protected by glycerol [35].

Collectively, the survival rate did not continue to increase with higher concentrations of cryoprotectants, indicating that both too low and too high concentrations were less effective. The optimal cryoprotectant combinations for each strain were identified. For *L. plantarum*, the optimal combination was 120 mg/mL of skim milk powder, 60 mg/mL of sucrose, and 80 mg/mL of L-proline. For *B. subtilis*, the optimal combination was 120 mg/mL of skim milk powder, 40 mg/mL of sucrose, 80 mg/mL of glycerol, and 60 mg/mL of L-proline.

### 3.2. Analysis of Response Surface Methodology to Determine the Optimum Concentration of Different Cryoprotectants

#### 3.2.1. Modelling of Response Surfaces

Table 3 and Table 4 show that both the cryoprotectant optimization models for *L. plantarum* (ML) and *B. subtilis* (MB) were highly significant (*p* < 0.01), with the misfit terms being non-significant (*p* > 0.05). This indicated that the models were well fitted and that unknown factors had minimal interference. The corrected coefficients of determination (R^2^) for the models were 0.9347 for *L. plantarum* and 0.9648 for *B. subtilis*, suggesting that 93.47% and 96.48% of the variation in response values could be explained by the models, with minimal experimental errors. These results demonstrated that the models were effective for analyzing and predicting the lyophilization survival of the strains under different cryoprotectant combinations.

Table 5 provides a detailed breakdown of the main effects, interaction effects, and quadratic effects of the tested factors on the response variable. The optimized quadratic polynomial equations for the cryoprotectants of *L. plantarum* and *B. subtilis* were derived through regression fitting as follows:

ML: Y = 91.40 + 2.01A_1_ + 0.55B_1_ + 5.81C_1_ − 2.13A_1_B_1_ − 4.09A_1_C_1_ − 6.45B_1_C_1_ − 10.93A_1_^2^ − 0.90B_1_^2^ − 13.77C_1_^2^ (3)

MB: Y = 81.42 − 1.98A_2_ + 2.83B_2_ + 1.01C_2_ + 4.14A_2_B_2_ − 3.38A_2_C_2_ + 7.43B_2_C_2_ − 12.71A_2_^2^ − 12.04B_2_^2^ − 7.39C_2_^2^ (4)

The influence of different cryoprotectants on strain survival, as indicated by the *p*-values, shows the following order of effectiveness: In the ML model, L-proline > skim milk powder > sucrose; in the MB model, sucrose > skim milk powder > glycerol.

#### 3.2.2. Response Surface Interaction Analysis

**Figure 2:**
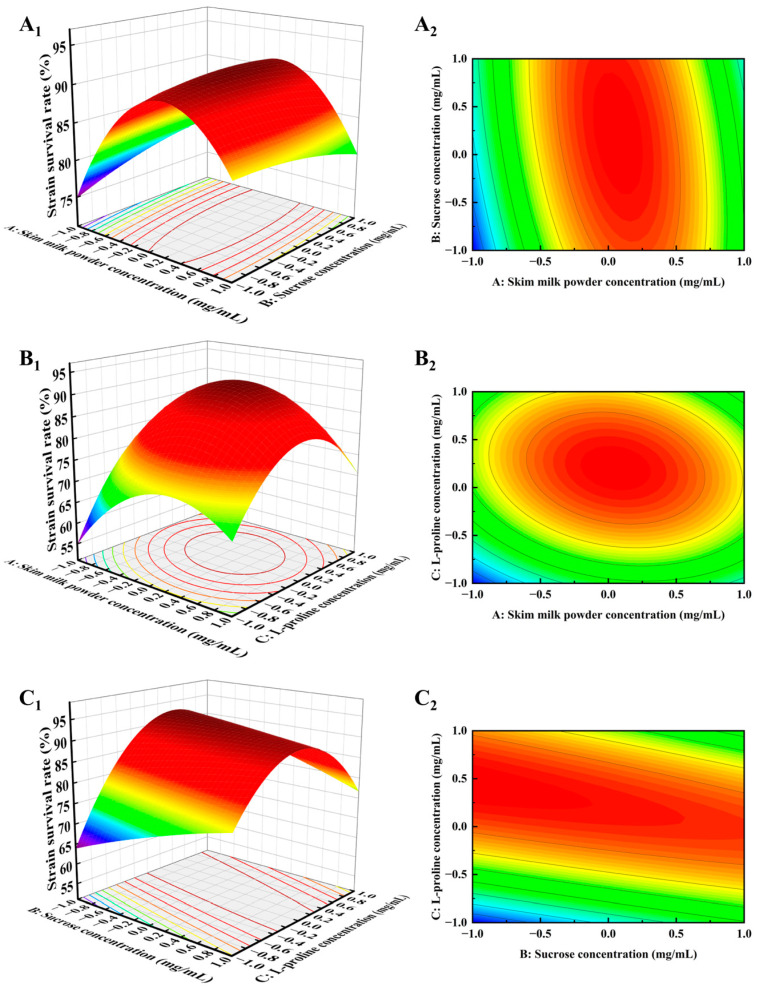
Response surface plots (**A_1_**–**C_1_**) and contour plots (**A_2_**–**C_2_**) of the two-way interaction of skim milk powder, sucrose, and L-proline for the response surface model *L. plantarum*.

The response values and interactions of the factors are represented by the response surface and contour plots. These plots allow for the evaluation of the interaction effects of the independent factors, with the shape of the contour lines visually indicating the strength of the interaction. A circular contour suggests minimal interaction, while an elliptical shape indicates a more pronounced interaction [36]. The interaction effects of the three cryoprotectants on lyophilization survival were analyzed using response surface and contour plots. Figure 2 illustrates the influence of the interaction among skim milk powder, sucrose, and L-proline on the freeze-dried survival rate of *L. plantarum*. In Figure 2A_2_, sucrose had a minimal effect on survival when the concentration of skim milk powder was at the optimal level. However, when sucrose was at its optimal concentration, strain survival varied significantly with changes in skim milk powder concentration, suggesting that skim milk powder had a greater impact on freeze-drying survival than sucrose.

Figure 2B_2_ further reveals that when the concentration of skim milk powder was at the optimal level, the contour lines representing survival rate variation with L-proline were denser. In contrast, when L-proline concentration was held constant, the contours for survival rate variation with skim milk powder were more spread out, indicating that L-proline had a stronger effect than skim milk powder. These observations confirmed that, in order of significance, the factors influencing *L. plantarum* survival were L-proline, skim milk powder, and sucrose, which aligned with the *p*-value analysis (Table 3).

**Figure 3:**
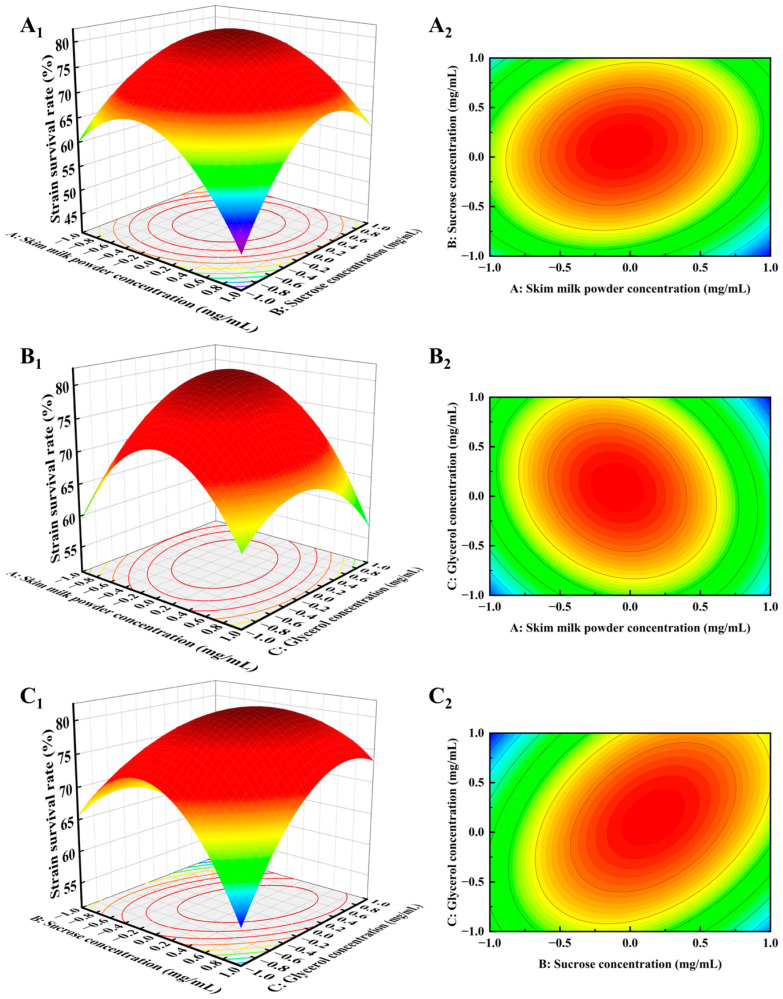
Response surface plots (**A_1_**–**C_1_**) and contour plots (**A_2_**–**C*_2_***) of the two-way interactions between skim milk powder, sucrose, and glycerol for the response surface model *B. subtilis*.

Figure 3 examines the interactions between skim milk powder (A), sucrose (B), and glycerol (C) and their effects on the freeze-dried survival of *B. subtilis*. In Figure 3A_2_, the contour lines for sucrose showed a higher density along the axis compared to skim milk powder, indicating that sucrose had a greater impact on *B. subtilis* survival. Figure 3B_2_ further demonstrates that the contour lines for skim milk powder were denser than those for glycerol, highlighting that the concentration of skim milk powder had a more significant effect on *B. subtilis* survival than glycerol. Thus, the order of influence for *B. subtilis* was sucrose, skim milk powder, and glycerol, consistent with the *p*-value results (Table 4). These findings align with a previous study [32], which reported that skim milk powder had a more significant effect on strain survival than glycerol when optimizing a lyophilized protectant for *Lactobacillus rhamnosus* using response surface methodology (RSM).

### 3.3. Analysis of Validation Experiment

The optimal cryoprotectant parameters were determined through numerical analysis using Design-Expert 8, with the objective of maximizing the survival of direct vat set (DVS) starters. For *L. plantarum*, the optimal cryoprotectant formulation was 122.2 mg/mL of skim milk powder, 50 mg/mL of sucrose, and 84.3 mg/mL of L-proline, resulting in a predicted maximum survival of 92.81%. For *B. subtilis*, the optimal formulation was 118.6 mg/mL of skim milk powder, 41.6 mg/mL of sucrose, 81.6 mg/mL of glycerol, and 60 mg/mL of L-proline, with a predicted maximum survival of 81.79%. To validate these predictions, experimental trials were conducted under the optimized conditions. The observed survival rates were 91.74 ± 2.00% for *L. plantarum* and 80.67 ± 3.31% for *B. subtilis*, which were consistent with the model predictions, demonstrating the reliability and feasibility of using RSM for optimizing cryoprotectant formulations. In comparison, a study [30] optimized cryoprotectants for *L. plantarum* LB12 using skim milk powder, monosodium glutamate, and alginate, achieving a survival rate of 92.87 ± 1.25%. Similarly, He et al. [37] optimized cryoprotectants for *B. subtilis* natto powder using an orthogonal array, resulting in a survival rate of 77.00%. These findings, together with the present study, confirmed that the combination of skim milk powder, sucrose, L-proline, and glycerol offered significant protective effects for both *L. plantarum* and *B. subtilis*.

### 3.4. Effects of Temperature on the Survival Rate of the Direct Vat Set Starter During Storage

**Figure 4:**
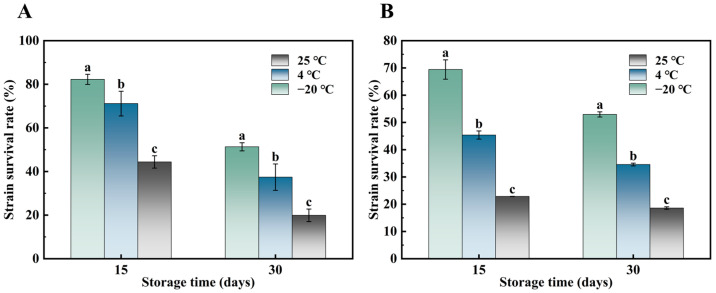
Survival rates of *L. plantarum* (**A**) powder and *B. subtilis* (**B**) powder at different storage temperatures. For the same storage time, different letters indicate that the corresponding values at different temperatures were significantly different (*p* < 0.05).

Cell viability during storage in a dry state is primarily influenced by storage temperature, humidity, water activity, and the presence of oxygen [38]. Celik et al. [39] studied the storage stability of *Bifidobacterium bifidum* after freeze-drying and identified optimal storage conditions, including refrigeration, using nitrogen instead of oxygen, and a moisture activity range of 0.11 to 0.22. In this study, storage temperature was the key variable. As shown in Figure 4, the survival rates of *L. plantarum* and *B. subtilis* decreased over time at 25 °C, 4 °C, and −20 °C. The highest survival rates were observed at −20 °C, with *L. plantarum* and *B. subtilis* surviving at 51.33% and 52.89%, respectively, after 30 days of storage. In contrast, the survival rate decreased most rapidly at 25 °C. These results indicate that −20 °C was the most effective storage temperature for preserving the strains, which is consistent with previous findings [40]. However, storage at −20 °C in large-scale production or distribution may present economic challenges, requiring further research into alternative preservation strategies such as optimizing packaging methods and materials to improve cell survival during storage [41].

### 3.5. Fermentation Capacity of the Direct Vat Set Starter in Sichuan Paocai

To verify the fermentation capacity of DVS starters for Sichuan paocai production, we compared the Sichuan paocai produced using DVS starters with the natural fermentation and direct complex fermentation with the two strains, and we investigated the pH, nitrites, and sensory properties of the fermented Sichuan paocai.

**Figure 5:**
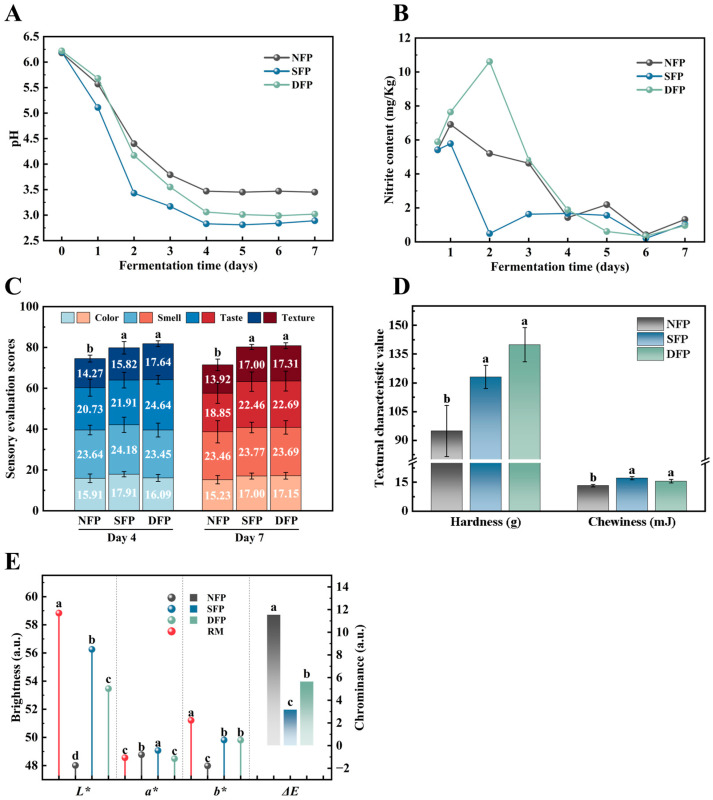
Physicochemical indexes and sensory evaluation of Sichuan paocai made using three different fermentation methods. Changes in pH (**A**), changes in nitrite content (**B**), sensory scores on the fourth and seventh day (**C**), differences in texture and structure on the seventh day of fermentation (**D**), and chromaticity color differences between the three groups of paocai and radish raw material (RM) on the seventh day of fermentation (**E**). Values corresponding to different letters in the determination of different indicators were significantly different (*p* < 0.05).

pH value is a key indicator for the maturity of Sichuan paocai [42]. As depicted in Figure 5A, the initial pH of the paocai solutions was 6.20, and by the 3rd day of fermentation, the pH in all groups had dropped below 4.00, indicating that the fermentation was becoming mature. From day 4 onward, the pH stabilized in all three groups, with the inoculated fermentation group (SFP) and the DVS starter fermentation group (DFP) showing significantly lower pH values than the natural fermentation group (NFP). The pH of the DFP group decreased more slowly than the NFP and SFP groups on day 1, but it dropped sharply thereafter. This could be due to the metabolite action of *B. subtilis* during the early stage of fermentation. *B. subtilis* can secrete proteases to metabolize proteins and amino acids, producing ammonia or alkaline by-products [43], thus increasing the pH value. The results of the growth property of *B. subtilis* in this study further confirmed that the pH value of the culture medium increased after the logarithmic growth phase (Figure S1B,D). In contrast, *L. plantarum* had a slower growth rate (Figure S1A,C), leading to the domination of *B. subtilis* after DVS starter addition in the early fermentation stage of paocai. However, after day 1, the vigorous anaerobic metabolism of *L. plantarum* accelerated the fermentation of the DFP group, leading to a faster decrease in pH that mirrored the SFP group’s trend (Figure 5A).

Nitrites can combine with protein breakdown products to form carcinogenic N-nitroso compounds [44], threatening the health of consumers. Nitrites are mainly produced by the action of nitrate-reducing bacteria during vegetable fermentation [45]. Therefore, nitrite content must be monitored to ensure paocai safety. Figure 5B shows that nitrite peaks for SFP, NFP, and DFP increased sequentially, with the highest peak for DFP at 10.61 mg/kg. This could be due to the high metabolite activity of heterotrophic bacteria in the early fermentation stage. Additionally, these bacteria might secrete nitrate reductase, converting nitrate to nitrite in the vegetable raw material [46]. The DVS starters, containing skim milk powder, sucrose, glycerol, and L-proline, could promote the growth of these heterotrophic bacteria. In addition, *L. plantarum* grew more slowly than *B. subtilis* in the early fermentation stage, leading to a higher rate of nitrite production than degradation, thus increasing nitrite levels in the DFP group compared to the SFP and NFP groups. Of note, the nitrite content of the three groups was well below the standard limit of 20 mg/kg for pickled vegetables [47], demonstrating that the DVS starters containing *B. subtilis* and *L. plantarum* could be put into application to ensure the safety of Sichuan paocai.

Sensory evaluation is the assessment of the overall quality and consumer acceptance of Sichuan paocai through sensory indicators (e.g., color, aroma, taste, texture, etc.) [48]. All sensory scores (Figure 5C) revealed that both the SFP and DFP groups scored higher than the NFP group, and the scores were close to each other, suggesting that the cryoprotectants were effective in protecting the two strains, and fermentation vigor was not affected. On day 7, the sensory scores for SFP and DFP were similar, but DFP had better taste. This indicated that inoculated fermentation improved the quality of Sichuan paocai, and the strains had a consistent effect on its sensory acceptability. The texture, particularly the hardness and chewiness, was notably better in the SFP and DFP groups compared to the NFP group (Figure 5D), which was consistent with the sensory evaluation results (Figure 5C), suggesting that DVS starters enhanced the texture of the Sichuan paocai.

The color analysis showed a significant decrease in both L* and b* values, indicating that the Sichuan paocai became darker. The Δ*E* of the NFP, SFP, and DFP groups were 11.54, 3.17, and 5.65, respectively, indicating differences in the color stability among these groups. Since the clinically perceptible level of Δ*E* is between 1.00 and 3.70 [49], this suggests that the color changes due to the maturation of Sichuan paocai were noticeable to the human eye. The difference in the Δ*E* value might be caused by polyphenols being oxidized to form quinones, which polymerize into brown pigments [50]. It has been reported that oxalic acid and citric acid play an important role in inhibiting polyphenol oxidase [51], and we previously found that *B. subtilis* Y61 inoculation in Sichuan paocai led to significantly higher organic acid contents compared to natural fermentation [14], resulting in the suppression of browning.

## 4. Conclusions

In a previous study, the feasibility of using *Bacillus subtilis* Y61 in the fermentation of Sichuan paocai was established, along with the optimal ratio of *B. subtilis* Y61 combined with *Lactiplantibacillus plantarum* for fermentation. To develop DVS starters with high viability and viable counts, cryoprotectants for *L. plantarum* and *B. subtilis* were optimized using a response surface methodology. The observed survival rates of the two strains closely aligned with the predicted values. Storage experiments showed that the DVS starters performed best at lower temperatures, with strain survival rates evaluated on the 15th and 30th day. When the optimized DVS starters were applied to Sichuan paocai fermentation, the product exhibited characteristics similar to Sichuan paocai fermented with inoculated strains prior to freeze-drying. Furthermore, the color, flavor, and overall organoleptic quality of the fermented product were superior to that of naturally fermented Sichuan paocai. Overall, this study provides a theoretical foundation for the preparation and application of DVS starters containing *L. plantarum* and *B. subtilis* in Sichuan paocai production.

## Figures and Tables

**Table 1:** Different cryoprotectants and preparation concentrations.

Strain	Cryoprotectant Concentration (mg/mL)	Gradient					
		0	1	2	3	4	5
*L. plantarum* SICC 1.1418	Skim milk powder	0	40	80	120	160	200
	Sucrose	0	20	40	60	80	100
	L-proline	0	20	40	60	80	100
	Glycerol	0	20	40	60	80	100
*B. subtilis* Y61	Skim milk powder	0	40	80	120	160	200
	Sucrose	0	20	40	60	80	100
	L-proline	0	20	40	60	80	100
	Glycerol	0	20	40	60	80	100

**Table 2:** Box–Behnken experimental design and the results of the lyophilized survival rate.

Number	Factor Level			Survival Rate (%)	Factor Level			Survival Rate (%)
	A_1_ (mg/mL)	B_1_ (mg/mL)	C_1_ (mg/mL)	*L. plantarum* SICC 1.1418	A_2_ (mg/mL)	B_2_ (mg/mL)	C_2_ (mg/mL)	*B. subtilis* Y61
1	−1 (100)	−1 (50)	0 (80)	78.82 ± 2.29	−1 (100)	−1 (30)	0 (80)	56.76 ± 2.64
2	1 (140)	0 (60)	1 (90)	71.36 ± 1.84	0 (120)	0 (40)	0 (80)	79.05 ± 0.89
3	0 (120)	0 (60)	0 (80)	91.82 ± 2.41	0 (120)	1 (50)	−1 (70)	56.42 ± 1.79
4	0 (120)	0 (60)	0 (80)	87.00 ± 0.55	−1 (100)	0 (40)	1 (90)	70.95 ± 1.22
5	0 (120)	0 (60)	0 (80)	90.45 ± 0.79	−1 (100)	0 (40)	−1 (70)	59.12 ± 1.01
6	−1 (100)	0 (60)	−1 (70)	53.86 ± 1.76	−1 (100)	1 (50)	0 (80)	57.09 ± 3.00
7	0 (120)	−1 (50)	−1 (70)	60.91 ± 2.41	0 (120)	−1 (30)	−1 (70)	68.58 ± 0.68
8	0 (120)	0 (60)	0 (80)	94.55 ± 0.91	0 (120)	0 (40)	0 (80)	83.45 ± 0.59
9	0 (120)	−1 (50)	1 (90)	87.27 ± 0.91	0 (120)	0 (40)	0 (80)	83.78 ± 2.36
10	−1 (100)	0 (60)	1 (90)	71.82 ± 2.73	0 (120)	1 (50)	1 (90)	70.27 ± 2.22
11	1 (140)	0 (60)	−1 (70)	69.77 ± 2.19	1 (140)	0 (40)	1 (90)	56.76 ± 1.79
12	0 (120)	0 (60)	0 (80)	93.18 ± 1.98	0 (120)	−1 (30)	1 (90)	52.70 ± 2.36
13	−1 (100)	1 (70)	0 (80)	80.00 ± 0.91	1 (140)	1 (50)	0 (80)	64.86 ± 1.22
14	0 (120)	1 (70)	1 (90)	79.64 ± 3.34	1 (140)	0 (40)	−1 (70)	58.45 ± 1.35
15	1 (140)	1 (70)	0 (80)	76.06 ± 1.39	1 (140)	−1 (30)	0 (80)	47.97 ± 1.35
16	1 (140)	−1 (50)	0 (80)	83.41 ± 0.99	0 (120)	0 (40)	0 (80)	81.42 ± 0.68
17	0 (120)	1 (70)	−1 (70)	79.09 ± 0.00	0 (120)	0 (40)	0 (80)	79.39 ± 1.79

Note: −1, 0, 1 denote the low-level concentration, significant center concentration, and high-level concentration of the selected cryoprotectants, respectively. The specific concentrations of the three cryoprotectants to be optimized for both strains are shown in the brackets.

**Table 3:** Analysis of variance for *L. plantarum* Box–Benken design regression model.

Source of Variance	Sum of Squares	Degrees of Freedom	Mean Square	*F*-Value	*p*-Value	Significance
Model	1949.9	9	216.66	14.78	0.0009	**
A	32.4	1	32.4	2.21	0.1807	
B	2.4	1	2.4	0.16	0.698	
C	269.82	1	269.82	18.4	0.0036	*
AB	18.19	1	18.19	1.24	0.3021	
AC	66.99	1	66.99	4.57	0.0699	
BC	166.54	1	166.54	11.36	0.0119	*
A_2_	502.67	1	502.67	34.29	0.0006	**
B_2_	3.42	1	3.42	0.23	0.6438	
C_2_	798.52	1	798.52	54.47	0.0002	**
Residual	102.63	7	14.66			
Lack of fit	69.1	3	23.03	2.75	0.1768	
Pure error	33.53	4	8.38			
Cor total	2052.52	16				

Note: ** indicates highly significant difference (*p* < 0.01); * indicates significant difference (*p* < 0.05).

**Table 4:** Analysis of variance for *B. subtilis* Box–Benken design regression model.

Source of Variance	Sum of Squares	Degrees of Freedom	Mean Square	*F*-Value	*p*-Value	Significance
Model	2126.12	9	236.24	20.85	0.0003	**
A	31.52	1	31.52	2.78	0.1393	
B	64.01	1	64.01	5.65	0.0491	*
C	8.22	1	8.22	0.73	0.4225	
AB	68.56	1	68.56	6.05	0.0435	*
AC	45.7	1	45.7	4.03	0.0846	
BC	220.97	1	220.97	19.5	0.0031	**
A_2_	680.21	1	680.21	60.03	0.0001	**
B_2_	610.14	1	610.14	53.84	0.0002	**
C_2_	229.81	1	229.81	20.28	0.0028	**
Residual	79.32	7	11.33			
Lack of fit	59.89	3	19.96	4.11	0.1028	
Pure error	19.43	4	4.86			
Cor Total	2205.44	16				

Note: ** indicates highly significant difference (*p* < 0.01); * indicates significant difference (*p* < 0.05).

**Table 5:** Estimated effects and coefficients for the response surface model.

*L. plantarum* SICC 1.1418					*B. subtilis* Y61				
Factor	Coefficient Estimate	Standard Error	95% CI Low	95% CI High	Factor	Coefficient Estimate	Standard Error	95% CI Low	95% CI High
Intercept	91.40	1.71	87.35	95.45	Intercept	81.42	1.51	77.86	84.98
A_1_	2.01	1.35	−1.19	5.21	A_2_	−1.98	1.19	−4.80	0.83
B_1_	0.55	1.35	−2.65	3.75	B_2_	2.83	1.19	0.015	5.64
C_1_	5.81	1.35	2.61	9.01	C_2_	1.01	1.19	−1.80	3.83
A_1_B_1_	−2.13	1.91	−6.66	2.39	A_2_B_2_	4.14	1.68	0.16	8.12
A_1_C_1_	−4.09	1.91	−8.62	0.43	A_2_C_2_	−3.38	1.68	−7.36	0.60
B_1_C_1_	−6.45	1.91	−10.98	−1.93	B_2_C_2_	7.43	1.68	3.45	11.41
A_1_^2^	−10.93	1.87	−15.34	−6.51	A_2_^2^	−12.71	1.64	−16.59	−8.83
B_1_^2^	−0.90	1.87	−5.31	3.51	B_2_^2^	−12.04	1.64	−15.92	−8.16
C_1_^2^	−13.77	1.87	−18.18	−9.36	C_2_^2^	−7.39	1.64	−11.27	−3.51

Note: Confidence interval (CI).

## Data Availability

The original contributions presented in this study are included in the article and Supplementary Materials. Further inquiries can be directed to the corresponding author.

## Supplementary Materials

The following supporting information can be downloaded at: https://www.mdpi.com/article/10.3390/foods14020157/s1, Figure S1: Growth curves of *L. plantarum* and *B. subtilis* Y61; Figure S2: Physicochemical indexes and sensory evaluation of Sichuan paocai made by five different combinations of *L. plantarum* and *B. subtilis* Y61 (L:B = 3:1, L:B = 2:1, L:B = 1:1, L:B = 1:2 and L:B = 1:3) and the natural fermentation group (NFP); Table S1: Sensory evaluation criteria of Sichuan paocai.
