# Supplementary material for: Optimization of the Cryoprotectants for Direct Vat Set Starters in Sichuan Paocai Using Response Surface Methodology
Source: Foods. 2025 Jan 7;14(2):157. doi: 10.3390/foods14020157 (PMC11764757; doi:10.3390/foods14020157)
Supplement: Supplementary file 1 [file foods-14-00157-s001.zip › foods-3364935-supplementary.pdf]

Table S1. Sensory evaluation criteria of Sichuan paocai.

Evaluation indicators	Evaluation criteria	score
Color	The color is normal, the radish is fresh, and there is no mold bloom or floating film on the surface.	15~20
	The color is slightly yellow, the radish is relatively fresh, and there is a small amount of mold bloom and floating film on the surface.	8~14
	The color is yellowish and dull, the radish is not fresh, and there is a considerable amount of mold bloom and floating film on the surface.	0~7
Smell	The aroma is rich and pleasant, with no off-odors, carrying a fresh radish fragrance.	21~30
	The aroma is moderate, with no unpleasant odors.	11~20
	The aroma is weak, with unpleasant odors present.	0~10
Taste	The flavor is rich, the texture is smooth, and the balance of sour, salty, and spicy is just right.	21~30
	The flavor is average, either slightly too sour or lacking sourness, slightly too salty or lacking saltiness, with no off-flavors.	11~20
	The flavor is poor, either overly sour or not sour enough, overly salty or not salty enough, with unpleasant off-flavors.	0~10
Texture	The texture is just right, crisp and tasty.	15~20
	The texture is somewhat soft, with moderate crispness.	8~14
	The texture is overly soft, with no crispness.	0~7

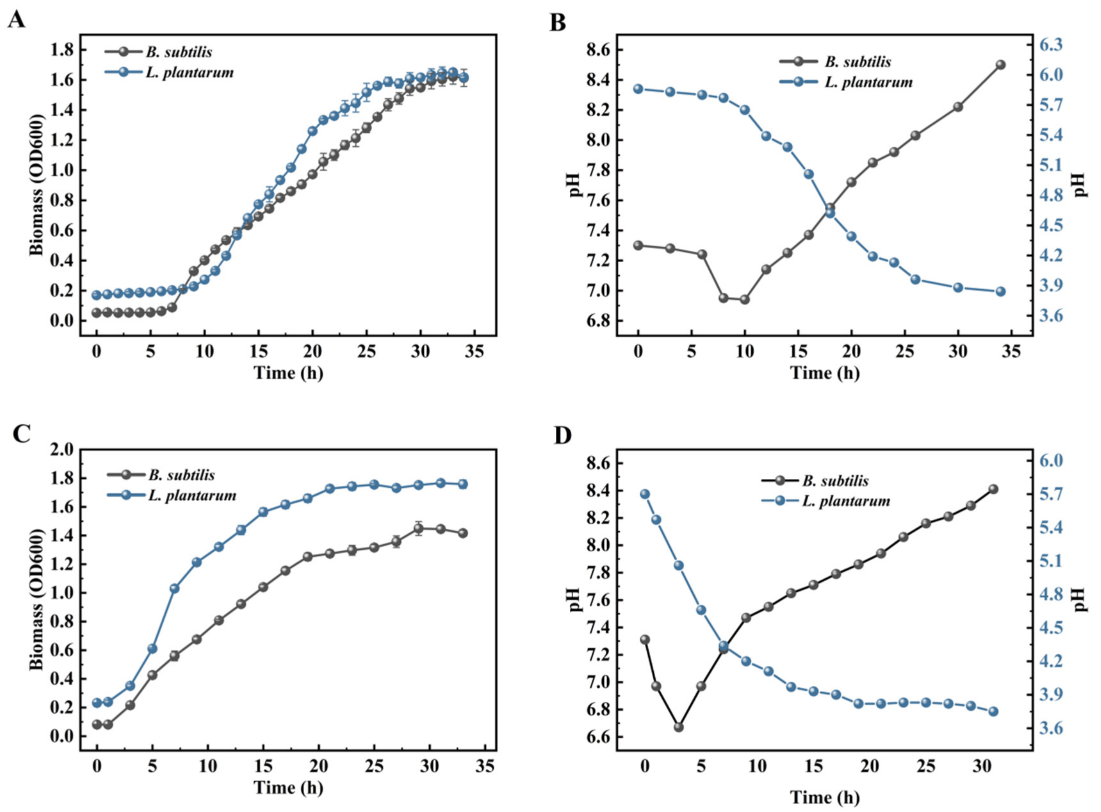

Figure S1. Growth curves of *L. plantarum* and *B. subtilis* Y61. Biomass (**A**) and pH (B) of the initial culture solution, and biomass (**C**) and pH (**D**) of the enriched culture solution.

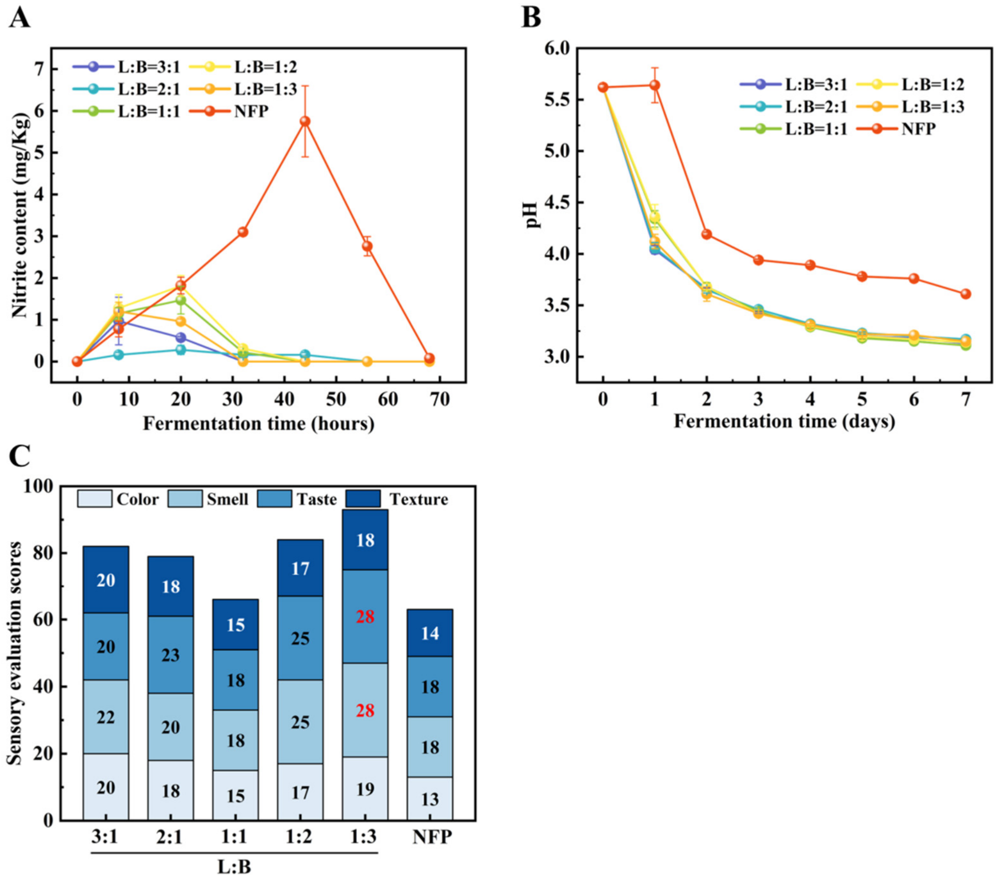

Figure S2. Physicochemical indexes and sensory evaluation of Sichuan paocai made by five different combinations of *L. plantarum* and *B. subtilis* Y61(L:B=3:1, L:B=2:1, L:B=1:1, L:B=1:2 and L:B=1:3) and natural fermentation group (NFP). Changes in nitrite content (**A**), changes in pH (**B**), sensory scores (**C**).
